# Fucoidan-Supplemented Diet Potentiates Immune Checkpoint Blockage by Enhancing Antitumor Immunity

**DOI:** 10.3389/fcell.2021.733246

**Published:** 2021-08-09

**Authors:** Juan Yang, Xianzhi Yang, Wenfeng Pan, Mingshuo Wang, Yuxiong Lu, Jianeng Zhang, Ziqian Fang, Xiaomin Zhang, Yin Ji, Jin-Xin Bei, Jiajun Dong, Yi Wu, Chaoyun Pan, Guangli Yu, Penghui Zhou, Bo Li

**Affiliations:** ^1^Jiangmen Central Hospital, Affiliated Jiangmen Hospital, Zhongshan School of Medicine, Sun Yat-sen University, Guangdong, China; ^2^State Key Laboratory of Oncology in South China, Collaborative Innovation Center for Cancer Medicine, Sun Yat-sen University Cancer Center, Guangdong, China; ^3^Clinical Biological Resource Bank, Guangzhou Institute of Pediatrics, Guangzhou Women and Children’s Hospital, Zhongshan School of Medicine, Sun Yat-sen University, Guangzhou, China; ^4^State Key Laboratory of Translational Medicine and Innovative Drug Development, Simcere Diagnostics Co., Ltd., Jiangsu, China; ^5^Center for Precision Medicine, Sun Yat-sen University, Guangdong, China; ^6^Key Laboratory of Marine Drugs of Ministry of Education, Shandong Provincial Key Laboratory of Glycoscience and Glycotechnology, School of Medicine and Pharmacy, Ocean University of China, Shandong, China; ^7^Laboratory for Marine Drugs and Bioproducts, Pilot National Laboratory for Marine Science and Technology (Qingdao), Shandong, China

**Keywords:** natural product, fucoidan, immunotherapy, cancer, T cells

## Abstract

Immune checkpoint blockade (ICB) therapies such as PD-1 antibodies have produced significant clinical responses in treating a variety of human malignancies, yet only a subset of cancer patients benefit from such therapy. To improve the ICB efficacy, combinations with additional therapeutics were under intensive investigation. Recently, special dietary compositions that can lower the cancer risk or inhibit cancer progression have drawn significant attention, although few were reported to show synergistic effects with ICB therapies. Interestingly, Fucoidan is naturally derived from edible brown algae and exhibits antitumor and immunomodulatory activities. Here we discover that fucoidan-supplemented diet significantly improves the antitumor activities of PD-1 antibodies *in vivo*. Specifically, fucoidan as a dietary ingredient strongly inhibits tumor growth when co-administrated with PD-1 antibodies, which effects can be further strengthened when fucoidan is applied before PD-1 treatments. Immune analysis revealed that fucoidan consistently promotes the activation of tumor-infiltrating CD8^+^ T cells, which support the evident synergies with ICB therapies. RNAseq analysis suggested that the JAK-STAT pathway is critical for fucoidan to enhance the effector function of CD8^+^ T cells, which could be otherwise attenuated by disruption of the T-cell receptor (TCR)/CD3 complex on the cell surface. Mechanistically, fucoidan interacts with this complex and augments TCR-mediated signaling that cooperate with the JAK-STAT pathway to stimulate T cell activation. Taken together, we demonstrated that fucoidan is a promising dietary supplement combined with ICB therapies to treat malignancies, and dissected an underappreciated mechanism for fucoidan-elicited immunomodulatory effects in cancer.

## Introduction

Immune checkpoint blockade (ICB) therapies have yielded appreciable clinical benefits in treating a variety of tumor types including melanoma (Pardoll, 2012; Li et al., 2016; Gong et al., 2018). Nivolumab and Pembrolizumab, two programmed death 1 (PD-1) antibodies approved by FDA, have been reported to significantly prolong progression-free and/or overall survival in patients with advanced melanoma (Eggermont et al., 2018; Eroglu et al., 2018), non-small cell lung cancer (Herbst et al., 2019), esophageal squamous-cell carcinoma (Kato et al., 2019), etc. Despite these impressive clinical effects, a great number of patients exhibit resistance or relapse after treatments (Minn and Wherry, 2016; Seidel et al., 2018). Therefore, there is an urgent need to improve the therapeutic efficacy by combining PD-1 antibodies with other therapeutics, such as tumor vaccines, oncolytic viruses, CTLA-4 antibodies, targeted therapies, radio- and chemo-therapies (Ott et al., 2017). In particular, the combination of two immunotherapies, CTLA-4 and PD-1 antibodies, exhibit enhanced efficacy against metastatic melanoma. However, the clinical incidence of immune-related adverse events (irAEs) is significantly higher with this combined approach (Kennedy and Salama, 2020). As such, alternative combination strategies with low side effects are under great demand to improve the therapeutic application of ICB.

Fucoidan, a fucose-enriched and sulfated polysaccharide molecule, is naturally derived from cell walls of edible brown algae and can be safely applied as a dietary supplement (Fitton, 2011; Citkowska et al., 2019). As a heterogeneous polysaccharide, the composition and structure of fucoidan vary widely in terms of seaweed species, growth environments, harvest seasons, and extraction methods (Kwak, 2014; Hsu and Hwang, 2019). Nevertheless, most fucoidan share common bioactivities including anti-cancer, anti-inflammatory, anti-bacterial, anti-viral, and anti-HIV activities. Among these functions, the anti-cancer properties of fucoidan attracted considerable attentions (Han et al., 2015; Atashrazm et al., 2016; Xue et al., 2020). Fucoidan has been observed to function as an antitumor agent in combination with conventional therapeutics in multiple models (Jin et al., 2014; Hsu and Hwang, 2019). However, the synergetic effects of fucoidan with immunotherapies have not been studied.

Using the murine melanoma model, we discovered that fucoidan-supplemented diet greatly improved the antitumor activities of PD-1 antibodies. If solely applied, however, fucoidan had no effect on melanoma cell proliferation and apoptosis *in vitro*, and failed to inhibit melanoma tumor growth *in vivo*. These results suggested that fucoidan synergizes with PD-1 antibodies to restrain tumor growth likely through immune modulation. Inspired by the increased T cell infiltration into tumor tissues upon fucoidan administration, we performed flow cytometry and RNA sequencing analysis on fucoidan-treated CD8^+^ T cells, and found that fucoidan stimulated the activation and propagation of CD8^+^ T cells. Mechanistically, fucoidan interacts with the T-cell receptor (TCR)/CD3 complex and potentiates its downstream JAK-STAT signaling, which is essential for T cell activation. Our findings provide new insight into the mechanism whereby dietary fucoidan strengthens the antitumor activity of PD-1 antibodies, and suggest a new combination approach that may potentiate the clinical effects of immunotherapies.

## Materials and Methods

### Cell Culture

B16 and 293T cells were cultured in DMEM supplemented with 10% fetal bovine serum and 100 μg/ml penicillin/streptomycin. Jurkat cells were cultured in RPIM-1640 supplemented with 10% fetal bovine serum and 100 μg/ml penicillin/streptomycin. Primary T cells isolated by negative selection were cultured in complete RPMI media (RPMI 1640, 10% FBS, 100 μg/ml penicillin/streptomycin, 1% MEM Non-Essential Amino Acids Solution, 0.05 mM 2-mercaptoethanol, 2 mM L-glutamine). All cells were maintained in an incubator with a humidified atmosphere of 5% CO_2_ at 37°C. Cells used in this study have been confirmed negative for Mycoplasma.

### Fucoidan Extraction and Component Analysis

Fucoidan A and F were respectively extracted from the brown algae *Ascophyllum nodosum* and *Fucus vesiculosus*. *Ascophyllum nodosum* was sourced from Chile. *Fucus vesiculosus* was purchased from QingDao Gather Great Ocean Algae Industry Group Co., Ltd (Qingdao, China). Fucoidan was extracted by acid extraction with heat in aqueous media. In brief, dried seaweeds were grounded and delipidated using 95% ethanol at 80°C for 4 h. After extraction with water at 80°C for 3 cycles of 3h, supernatant harvested by centrifugation were precipitated with 80% ethanol at 4°C overnight. After that, crude fucoidan precipitate was vacuum-dried and dissolved in distilled water, then further purified by removing alginate at pH 1.0. The resultant solution was centrifuged at 8000 rpm for 10 min, and supernatant containing fucoidan was adjusted to pH 7.5 and lyophilized after dialysis.

The sulfate content and monosaccharide composition were further analyzed. Briefly, fucoidan was degraded in 1M HCl at 110°C for 6 h, and mixed with isopycnic BaCl_2_-gelatin. Absorbance was then measured at 400 nm. The sulfate content was calculated according to the standard curve of Na_2_SO_4_. Monosaccharide composition was determined by a 1-phenyl-3-methyl-5-pyrazolone precolumn derivatization HPLC using an Eclipse XDB-C18 column (Agilent, Santa Clara, CA, USA). The fucoidan compositions are summarized in Table 1.

**TABLE 1:** Chemical composition of Fucoidan A and F.

**Fucoidan**	**Source**	**Sulfate content (%)**	**Molecular weight**	**Monosaccharide composition (%)**					
				**Man**	**GlcA**	**Glc**	**Gal**	**Xyl**	**Fuc**
Fucoidan A	*Ascophyllum nodosum*	22.7	210kDa	7.5	5.3	8.1	3.8	17.8	58.5
Fucoidan F	*Fucus vesiculosus*	26.3	610kDa	5.7	5.5	9.9	5.3	8.3	65.8

### Allograft Mouse Model

C57BL/6 mice were purchased from Beijing Vital River Laboratory Animal Technology (Beijing, China) and maintained under the specific-pathogen-free condition. C57BL/6 mice (6-8 weeks old) were subcutaneously inoculated with B16 cells (2 × 10^5^ cells per mouse) in the right lower abdomen area. After inoculation, mice were randomly divided into the following groups: PD-1 antibody treated only, fucoidan A-supplemented diet only, fucoidan F-supplemented diet only, PD-1 antibody combined with fucoidan A-supplemented diet, and PD-1 antibody combined with fucoidan F-supplemented diet. PD-1 antibody was administered intraperitoneally (i.p.) with 200 μg per mouse at day 7, 10, and 13 after tumor inoculation. Fucoidan supplementation were conducted by orally feeding the mice with 40 mg/ml fucoidan in sterile H_2_O, 200 μl per day from the inoculation day until the experiment endpoint. In fucoidan pretreatment model, mice were fed with fucoidan containing diet 24 days before tumor inoculation. The anti-mouse PD-1 antibody was purified from culture supernatants of corresponding hybridoma cells (clone G4), provided by Dr. Lieping Chen at Yale University. Tumors were measured every 3 days by an electronic caliper from roughly day 7 when tumors were established, and tumor volumes calculated using the equation (length × width^2^)/2. At the end point, tumors were surgically removed and weighed. Blood, tumor, spleen, and tumor-draining lymph node tissues were harvested for further analysis.

### Xenograft Mouse Model

BALB/C nude mice were purchased from GemPharmatech (Nanjing, China) and maintained under the specific-pathogen-free condition. B16 cells (2 × 10^5^ per mouse) were subcutaneously injected into BALB/C nude mice (aged 6-8 weeks). After tumor inoculation, mice were randomly divided into 2 groups: PD-1 antibody treated only and PD-1 antibody combined with fucoidan A-supplemented diet. Treatments were performed as previously described. Tumors were measured by an electronic caliper at day 8, 10, 12, and 14 after tumor inoculation. Mice were euthanized and tumors harvested at day 14.

### Analysis of Bone Marrow-Derived Dendritic Cells (BMDCs)

Bone marrow was isolated from C57BL/6 mice and treated with ACK lysis buffer to remove the erythrocytes, then washed with PBS. Cells were cultured in complete RPMI media (RPMI 1640, 10% FBS, 100 μg/ml penicillin/streptomycin, 1% MEM Non-Essential Amino Acids Solution, 0.05 mM 2-mercaptoethanol, 2 mM L-glutamine) supplemented with 20 ng/ml IL-4 and 20 ng/ml granulocyte-macrophage colony stimulating factor (GM-CSF) for 6 days. After that, cells were treated with different concentrations of fucoidan A or F for 24 h, and the expression of CD40, CD80, CD86, and MHC II were analyzed by flow cytometry.

### Isolation of Tumor-Infiltrating Lymphocytes

B16 melanomas were cut into small pieces in petri dishes containing 10 ml PBS supplemented with 2% FBS, and washed with PBS. Tumors were resuspended in 15 ml RPMI supplemented with 2% FBS, 50 U/ml collagenase type IV (Invitrogen), 20 U/ml DNase (Roche) and incubated at 37°C for 2 h. Processed tissues were further dissociated using a MACS Dissociator (Miltenyi Biotech). Suspensions were washed three times with PBS and passed through a 70 μm strainer. Lymphocytes were isolated by Ficoll density gradient centrifugation for further analysis.

### Flow Cytometry

Spleens and tumor-draining lymph nodes were harvested from mice and rubbed with the rough surface of a glass slide to obtain single cell suspension. Spleens were treated with ACK lysis buffer to lyse the erythrocytes. Suspensions of spleens and lymph nodes were passed through a 70 μm strainer, incubated with specific antibodies for 30 minutes at room temperature, washed with PBS, and then analyzed using a FACSAria (BD Biosciences) flow cytometer. Peripheral blood was directly incubated with specific antibodies after processed with ACK lysis buffer. Fluorescence-conjugated anti-CD3, anti-CD19, anti-CD4, anti-CD8, anti-NK1.1, anti-CD11C, anti-CD40, anti-CD80, anti-CD86, and anti-MHC II were purchased from Biolegend.

### Apoptosis Assay

Cells exposed to 100 μg/ml fucoidan for 48 h were harvested, incubated with Annexin V for 20 min at room temperature, subsequently with propidium iodide (PI) (Invitrogen) for another 20 min, and then analyzed by a flow cytometer.

### Cell Cycle and Proliferation Assay

Cells were seeded in 6 well-plates and treated with 100 μg/ml fucoidan for 48 h. Then cells were collected, washed with cold PBS, and fixed with pre-cold 70% ethanol overnight at 4°C, followed by incubation with propidium iodide (PI) (Invitrogen) for 30 min shielded from light and analyzed by flow cytometry. Cell proliferation was determined using the CCK8 assay (Roche). Briefly, cells were seeded at 2 × 10^3^ cells/well in 96-well plates. Cells were cultured overnight to adhere, then incubated in 100 μl fresh medium containing various concentrations of fucoidan. At day 3, 10 μl CCK8 solution was added to each well, and incubated for 4 hours. Absorbance was measured at 450 nm.

### T Cell Activation Assay

Primary CD8^+^ T cells isolated by negative selection (Biolegend) from lymph nodes and spleens of C57BL/6 mouse were stimulated by immobilized anti-mouse CD3 and CD28 antibodies (3 μg/ml) in 96-well plate for 48 h. T cell activation and proliferation was indicated by CFSE staining.

### Cytokine Analysis

Intracellular cytokine staining was performed according to the manufacturer’s instructions (BD Bioscience). In brief, primary T and Jurkat cells were collected after stimulation for 48 h with anti-CD3 and anti-CD8. Cells were fixed, permeabilized and stained with antibodies specific for IFNγ, TNFα, or Granzyme B (Biolegend).

### RNAseq Analysis

CD8^+^ T cells activated by CD3/28 antibodies were divided into three groups: control group treated with PBS, fucoidan A group incubated with 10 μg/ml fucoidan A, and fucoidan F group incubated with 10 μg/ml fucoidan F. Total RNA was extracted with the TRIzol reagent (Invitrogen) according to the manufacturer’s instructions. The quality of total RNA was tested (RNA integrity number ≥9.5) for construction of sequencing libraries. After mRNA capture, fragmentation, reverse transcription, terminal repair, linker ligation and PCR amplification, second-generation sequencing was performed according to Illumina’s standard protocol. The sequencing results were further analyzed by gene set enrichment analysis (GSEA4.1). Genes with a ratio equal or greater than 2 were considered different. The R programming language was used to obtain the gene ontology category after running with Metascape^1^.

### ShRNA Interference

The shRNA oligoes of human CD3E were synthesized by BGI Tech (Guangzhou, China). ShRNA oligoes were cloned into a lentiviral vector with the miR30 backbone containing a U6 promotor. Lentiviral particles were produced from 293T cells transfected with the shRNA vector together with packaging plasmids. The shRNA sequences used in this study were designed as follows:

Human shCD3E-1

Forward: 5′-CCCCTGGTATTACACAGACACCATATAAC TGTGACATGTCAAAAATTATATGGTGTCTGTGTAATAC CT-3′

Reverse: 5′-CACCAGGTATTACACAGACACCATATAATTT TTTGACATGCACAGTTATATGGTGTCTGTGTAATACCA-3′

Human shCD3E-2

Forward: 5′-CCCCTCATCTCTGGAACCACAGTAATATC TGTGACATGTCAAAAAATATTACTGTGGTTCCAGAGAT GT-3′

Reverse: 5′-CACCACATCTCTGGAACCACAGTAATATTT TTTGACATGTCACAGATATTACTGTGGTTCCAGAGAT GA-3′

### Quantitative Reverse Transcription PCR

Total RNA was obtained from cells using TRIzol (Invitrogen) and reverse transcribed with the EasyScript One-Step gDNA Removal and cDNA Synthesis SuperMix (TransGen Biotech, AE311). The cDNA product was amplified with the SYBR Green qPCR MIX (TransGen Biotech) and a Biorad CFX96 Real-Time system according to the manufacturer’s instructions. Relative gene expression was normalized to glyceraldehyde-3-phosphate (GAPDH)/actin beta (ACTB) and calculated by 2^–ΔΔ*CT*^. The sequence of specific primers used in this study were designed as follows:

Human CD3E Forward: 5′-GTAGTAAGTCTGCTGGCC TCC-3′

Reverse: 5′-CCCCAAACGCCAACTGATAA-3′

Human GAPDH Forward: 5′-GTCTCCTCTGACTTCAAC AGCG-3′

Reverse: 5′-ACCACCCTGTTGCTGTAGCCAA-3′

Human GMZB Forward: 5′-CGACAGTACCATTGAGTTGT GCG-3′

Reverse: 5′-TTCGTCCATAGGAGACAATGCCC-3′

Human IL2 Forward: 5′-AGAACTCAAACCTCTGGAGG AAG-3′

Reverse: 5′-GCTGTCTCATCAGCATATTCACAC-3′

Mouse ACTB Forward: 5′-CATTGCTGACAGGATGCAGA AGG-3′

Reverse: 5′-TGCTGGAAGGTGGACAGTGAGG-3′

Mouse CD70 Forward: 5′-GCGGACTACTCAGTAA GCAGCA-3′

Reverse: 5′-TGTGAAGGACCTTCCCAAGGCT-3′

Mouse IL6 Forward: 5′-TACCACTTCACAAGTCGG AGGC-3′

Reverse: 5′-CTGCAAGTGCATCATCGTTGTTC-3′

Mouse CSF2 Forward: 5′-AACCTCCTGGATGACATG CCTG-3′

Reverse: 5′-AAATTGCCCCGTAGACCCTGCT-3′

Mouse IL3 Forward: 5′-CCTGCCTACATCTGCGAAT GAC-3′

Reverse: 5′-GAGGTTAGCACTGTCTCCAGATC-3′

Mouse IL23A Forward: 5′-CATGCTAGCCTGGAACGCA CAT-3′

Reverse: 5′-ACTGGCTGTTGTCCTTGAGTCC-3′

Mouse IL13 Forward: 5′-AACGGCAGCATGGTATGGA GTG-3′

Reverse: 5′-TGGGTCCTGTAGATGGCATTGC-3′

Mouse IL24 Forward: 5′-CGGCTTCACTTTAGGACCC TAG-3′

Reverse: 5′-CCCAAATCGGAACTCTTGACCC-3′

### Western Blot

Cells were lysed with RIPA lysis buffer supplemented with 1x protease and phosphatase inhibitor. After quantified by the Pierce^TM^ BCA Protein Assay Kit (Thermo Scientific), proteins were separated by electrophoresis in a 12% sodium dodecyl sulfate-polyacrylamide gel (SDS-PAGE) (Bio-Rad, Hercules, CA) and electrophoretically transferred onto a PVDF membrane (Amersham Pharmacia Biotech, Piscataway, NJ). CD3E was detected with CD3E (CD3-12) rat mAb (CST Signaling, cat. #4443) at 1:1000 dilution and goat anti-rat IgG (H + L) HRP conjugates (Proteintech, cat. #SA00001) at 1:2000 dilution. The protein bands were visualized by chemiluminescent HRP substrates (Millipore).

### FITC-UEA-I Staining

CD8^+^ T cells isolated by negative selection were cultured in anti-CD3/CD28-coating 24-well plates with fucoidan A or F incubation at 100 μg/ml for 24 h. Cells were then collected, washed with PBS, and stained with FITC Ulex Europaeus Agglutinin I (FITC-UEA-I) (1:100 dilution) for 1.5 h at room temperature.

### Confocal Microscopy

The overexpression of RFP-conjugated human CD3E (CD3E-RFP) in Jurkat cells were obtained by lentivirus infection. The human CD3E full-length sequence was amplified by PCR using Jurkat cDNA as template, and cloned into a lentiviral vector with the P2A-RFP cassette. The sequence of cloning primers were designed as follows:

CD3E-F: 5′-AAAAAGGATCCATGCAGTCGGGCA CTCA CTG-3′

CD3E-R: 5′-AAAAAGCTAGCGATGCGTCTCTGATTCA GGCC-3′

After overexpression, cells were cultured with fucoidan A or F at 100 μg/ml overnight, washed with PBS, and stained with FITC-UEA-I (1:100 dilution) for 1.5 h at room temperature. Nuclear counterstaining was performed with Hoechst 33342(1:5000) for 15 min at room temperature. Cells were then transferred into confocal dishes and analyzed by a Fast Airyscan LSM880 Confocal microscope (Zeiss) with 60x oil objective.

### Pull-Down Assay

Jurkat or isolated CD8^+^ T cells were collected and lysed with the Pierce IP lysis buffer (Thermo scientific) on ice for 30 min. Proteins lysates were then incubated with fucoidan for 8 h, and Ulex Europaeus Agglutinin I (UEA-I) conjugated agarose (Fisher scientific) overnight at 4°C under agitation, and then analyzed by immunoblot analysis.

### Statistical Analysis

Statistical analysis was performed with two-tailed unpaired *t*-tests. All error bars displayed within figures indicate the mean of distribution and represent the standard error of the mean (SEM) unless otherwise stated. *P* < 0.05 was considered statistically significant and P values were indicated by asterisks as followed: ^∗^*P* < 0.05, ^∗∗^*P* < 0.01, ^∗∗∗^*P* < 0.001, and n.s., non-significant.

## Results

### Fucoidan Diet Enhances the Antitumor Efficacy of PD-1 Antibodies

**FIGURE 1:**
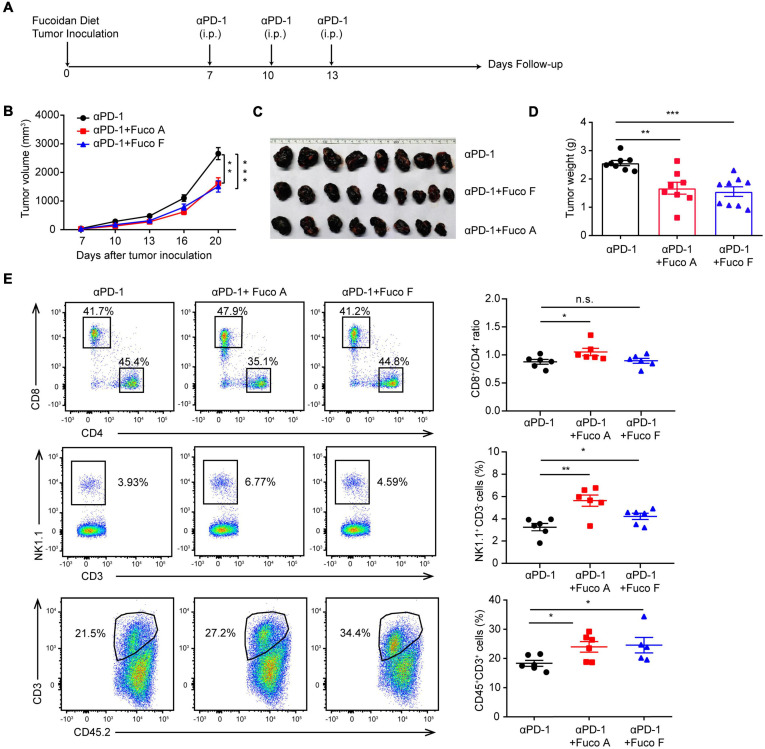
Fucoidan diet enhances the antitumor efficacy of PD-1 antibodies. **(A)** Experimental scheme for fucoidan-supplemented diet combined with PD-1 immunotherapy in the B16 melanoma model. C57BL/6 mice were subcutaneously inoculated with B16 cells, fed with fucoidan A or F from the inoculation day (day 0) to the end point (day 20). Each mouse was administered with 200 μg PD-1 antibody at day 7, 10, and 13. i.p., intraperitoneally. **(B)** Tumor volumes recorded at indicated times are shown. **(C)** Tumor images and **(D)** weights of harvested tumors at day 20 are shown. **(E)** Flow cytometry analysis of spleen resident and tumor infiltrating lymphocytes (TILs). Spleens, tumor-draining lymph nodes, and tumor tissues were collected respectively for flow cytometry analysis. Top: Representative FCM plots (left) and analysis (right) of CD8^+^ and CD4^+^ T cells in spleen. Middle: Representative FCM plots (left) and analysis (right) of NK cells in spleen. Bottom: FCM plots (left) and analysis (right) of CD45^+^CD3^+^ TILs. A, B, C, D (*n* = 8 mice per group) and E (*n* = 6 mice per group) are representative of two independent experiments. Fuco, fucoidan. Error bars, mean ± SEM. *P < 0.05, **P < 0.01, ***P < 0.001. n.s., non-significant.

To examine whether fucoidan-supplemented diet has a potentially synergistic effect with PD-1 immunotherapy, we subcutaneously inoculated B16 melanoma cells into C57BL/6 mice. Mice were treated with PD-1 antibodies in the presence or absence of fucoidan-supplemented diet (Figure 1A). Two unfractionated fucoidan species, namely fucoidan A and fucoidan F, were used throughout this study. They were respectively extracted from the brown algae *Ascophyllum nodosum* and *Fucus vesiculosus*, and subjected to composition analysis as previously described (Shang et al., 2016; Wang et al., 2019a; Table 1). We measured the growth of subcutaneous tumors and found that the combination of fucoidan diet and anti-PD-1 therapy resulted in synergistic antitumor responses. Compared with the anti-PD-1 treatment group, the tumor growth of mice was significantly slower in the combined treatment group (Figure 1B). Moreover, the tumor volumes and weights of combined treatment group were also markedly decreased (Figures 1C,D). To further investigate the potential immune changes, we performed flow cytometry analysis and found that mice receiving combined treatment exhibited increased population of CD8^+^ T cells in their spleen, especially in the case of fucoidan A (Figure 1E, upper part). In addition, fucoidan co-treated mice displayed an increased percentage of natural killer (NK) cells in their spleen (Figure 1E, middle part), and a concomitant increase of tumor infiltrating T cells (Figure 1E, bottom part). The percentage of NK and T cells in blood and lymph node have no obvious variation. These results indicate that fucoidan-supplemented diet is capable of reducing the growth of melanoma tumors when combined with PD-1 antibodies, demonstrating that fucoidan is a promising dietary ingredient to enhance the therapeutic efficacy of immunotherapy.

### Fucoidan Alone Is Insufficient to Inhibit Melanoma Cell Growth

**FIGURE 2:**
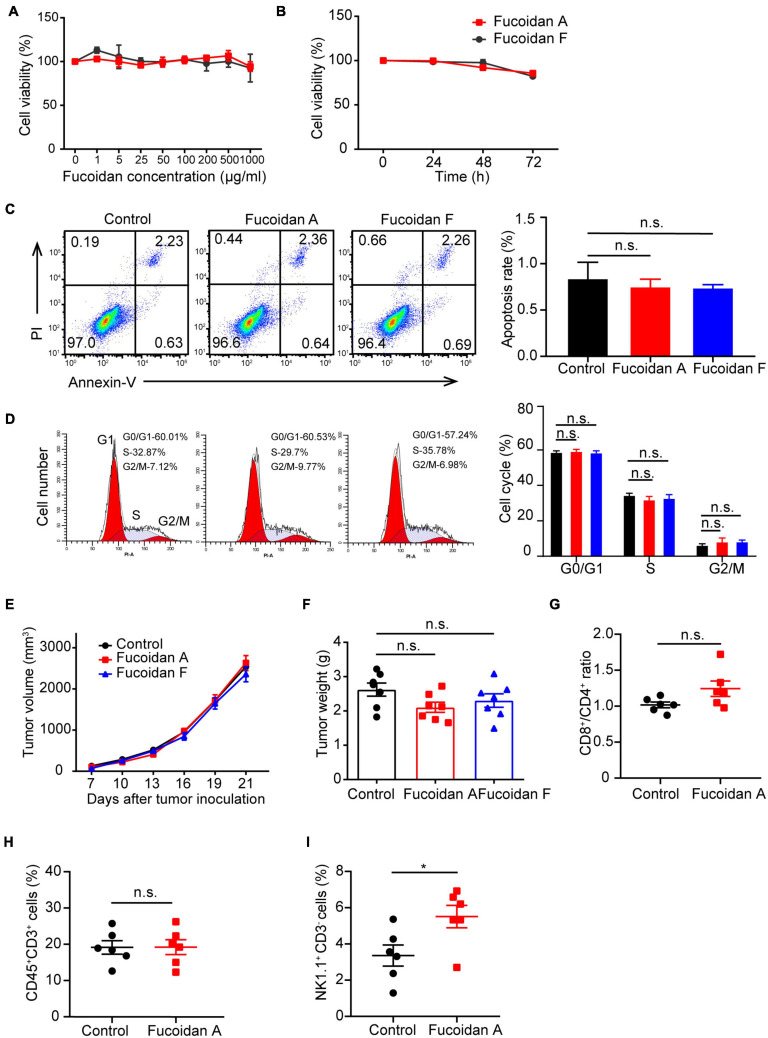
Fucoidan alone is insufficient to inhibit melanoma cell growth. **(A)** B16 cells were cultured with fucoidan A or F with indicated concentration for 48 h in 96-well plates. CCK8 assay was used to monitor cell proliferation. **(B)** B16 cells were cultured with 100 μg/ml fucoidan A or F for different durations. Cell apoptosis **(C)** was assessed by Annexin V/PI staining and cell cycle **(D)** was measured using PI staining. B16 cells were incubated with 100 μg/ml fucoidan A or F for 48 hours. Growth **(E)** and weights **(F)** of B16 tumors harvested from control mice, or mice with dietary fucoidan A or F treatments. The flow cytometry analysis of splenic CD8^+^/CD4^+^ T cells **(G)**, CD3^+^ TILs **(H)**, or splenic NK cells **(I)**, in fucoidan A treated or untreated B16-bearing mice. *In vitro* experiments were repeated at least three times. For *in vivo* experiments, data in E-F (*n* = 7 mice per group) and G-I (*n* = 6 mice per group) are representative of two independent replicates. Error bars, mean ± SEM. *P < 0.05, non-significant.

To explore whether fucoidan induces intrinsic antitumor responses in melanoma cells, we first investigated the effects of fucoidan on melanoma cell growth *in vitro*. B16 cells were incubated with different doses of fucoidan A or F for 48 h. CCK8 assays indicated that fucoidan had no measurable effects on the growth of B16 cells (Figure 2A). According to previous studies, 100 μg/ml is the half maximal inhibitory concentration of fucoidan against a variety of cancer cells (Yang et al., 2015; Atashrazm et al., 2016). With such dosage, B16 cell proliferation remained unaffected by fucoidan even increasing the incubation time to 72 h (Figure 2B). Since fucoidan have been reported to induce cell cycle arrest and interact with selective components of apoptotic pathway (Hyun et al., 2009; Kim et al., 2010; Park et al., 2017), we next examined the apoptotic index and cell cycle status of B16 cells after fucoidan treatments. Annexin-V/PI staining indicated that fucoidan failed to induce B16 cell apoptosis (Figure 2C). Similarly, B16 cell cycle was unaffected by fucoidan incubation (Figure 2D). These data suggested that fucoidan did not directly affect the growth or apoptosis of melanoma cells.

The next question is whether fucoidan alone is sufficient to confer immune remodelings that suppress tumor growth. To test this, we subcutaneously inoculated C57BL/6 mice with B16 cells and administrated dietary fucoidan in the absence of PD-1 antibodies. We followed the growth of subcutaneous tumors and found that tumor growth was comparable between fucoidan treated group and control group (Figure 2E), and at the end point fucoidan failed to reduce tumor weight (Figure 2F). Notably, a trend with lower tumor weight was associated with the fucoidan A group, suggesting that fucoidan A may be a bit more effective that fucoidan F in this setting. However, even with fucoidan A, we detected no significant difference between the experimental groups regarding the population of CD4^+^/CD8^+^ T cells in spleen (Figure 2G), as well as the number of tumor infiltrating T lymphocytes (Figure 2H). Similar to the results of combination therapy, we observed substantial increases in splenic NK population after fucoidan A treatments (Figure 2I). This finding is consistent with previous studies that fucoidan itself can elicit immune responses of NK cells (Ale et al., 2011; Atashrazm et al., 2016). Together, these results indicated that dietary fucoidan used in this study was insufficient to inhibit melanoma growth as a monotherapy, and its synergistic effects with PD-1 antibodies may not attributed to its ability to activate NK cells.

### Fucoidan Differentially Activates Bone Marrow-Derived Dendritic Cells

**FIGURE 3:**
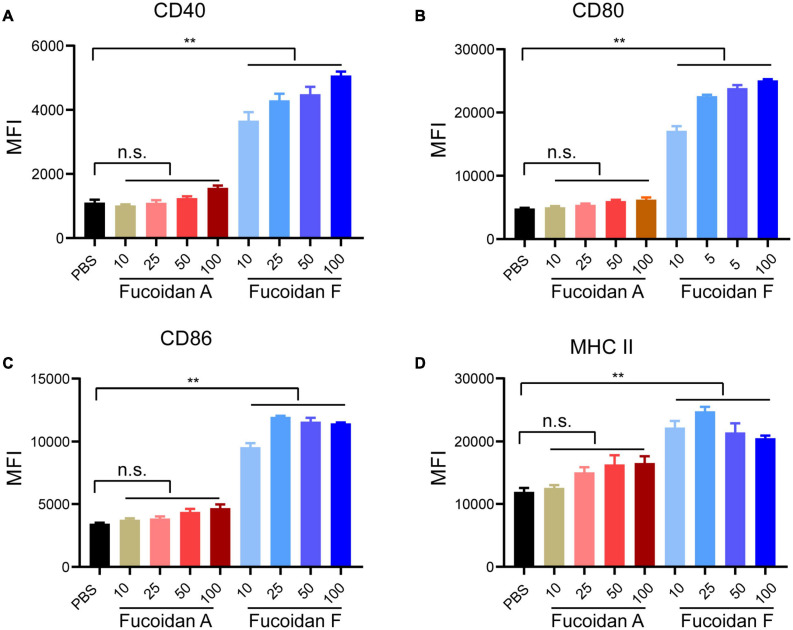
Fucoidan differentially activates bone marrow-derived dendritic cells. Bone marrow-derived monocytes from C57BL/6 mice were differentiated into DCs after incubation with IL4 and GM-CSF for 6 days. Immature DCs were then treated with indicated concentrations of fucoidan A or F for 24 h. Relative expression levels of CD40 **(A)**, CD80 **(B)**, CD86 **(C)**, and MHC II **(D)** quantified by mean fluorescence intensity (MFI) using flow cytometry, in DCs treated with fucoidan A or F with indicated concentrations (μg/ml). Error bars, mean ± SD. **P < 0.01. Data are representative of two independent experiments.

In addition to inducing NK activation, ample studies have established that fucoidan treatments alone promote the maturation of dendritic cells (DCs) through binding to toll-like receptors (TLR) and scavenger receptors-A (SR-A) on the surface of DCs (Jin et al., 2009; Makarenkova et al., 2012). To evaluate the possible effects of fucoidan A and F on DC maturation, we isolated monocytes from mouse bone marrows and incubated them with IL4 and GM-CSF to induce their differentiation to DCs, followed by fucoidan treatments. The expression levels of CD40, CD80, CD86, and MHC II, four conventional DC maturation markers, were all strongly upregulated in DCs upon fucoidan F treatments (Figures 3A-D). This is consistent with a plethora of studies demonstrating fucoidan-mediated DC activation. Interestingly, fucoidan F exhibited much stronger effects on DC maturation compared with fucoidan A, which are in sharp contrast with our previous observations that fucoidan A and F were equally effective in combination therapy with PD-1 antibodies (Figure 1B-D). These data suggested that fucoidan may contribute to synergistic tumor suppression by modulating immune populations other than NK and DC. Accordingly, the increased number of infiltrating T cells in co-treated tumors revealed possible functions of T cells in this context.

### Fucoidan Diet Enhances Antitumor Responses via Regulating T Cell Activities

**FIGURE 4:**
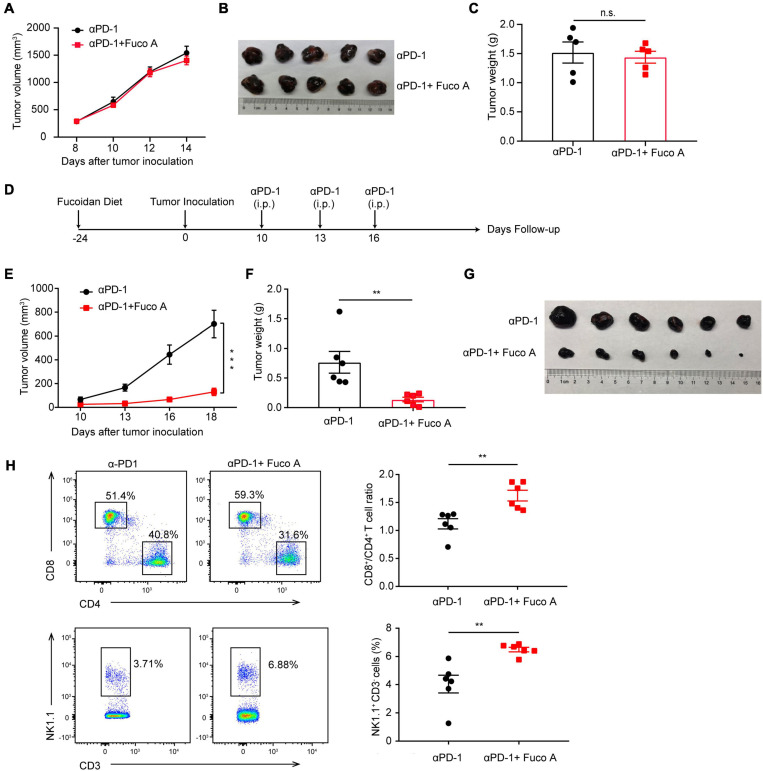
Fucoidan diet enhances antitumor responses via regulating T cell activities. **(A)** Tumor volumes of B16 melanoma from BALB/C nude mice. BALB/C nude mice were subcutaneously inoculated with B16 cells, fed with fucoidan A from the inoculation day (day 0) to the end point (day 14). Each mouse were administered with 200 μg PD-1 antibody at day 8, 10, and 12 (*n* = 5 mice per group). **(B)** Tumor pictures and **(C)** weights at day 14 were shown. **(D)** Experimental scheme for fucoidan pre-treatments combined with PD-1 immunotherapy in the B16 tumor model. C57BL/6 mice were fed with fucoidan A for 24 days prior to B16 inoculation. **(E)** Tumor volumes recorded at indicated times are shown. **(F)** Weights and **(G)** images of harvested tumors are shown (*n* = 6 mice per group). **(H)** Flow cytometry analysis of splenic CD8^+^, CD4^+^ T cells, and NK cells. FCM plots (left) and analysis (right) are representative of two independent experiments. Error bars, mean ± SEM. *P < 0.05, **P < 0.01, and n.s., non-significant.

To determine whether T cells are essential for the synergistic efficacy of fucoidan combined with PD-1 therapy, we utilized the BALB/C nude mouse model which is T-cell deficient. Briefly, we inoculated B16 cells into these mice and performed treatments according to the scheme as illustrated in Figure 1A. As expected, B16 melanoma growth remained unchanged irrespective of combination therapy (Figure 4A). The volumes (Figure 4B) and weights (Figure 4C) of formed tumors were comparable in mice receiving the fucoidan diet combined with PD-1 treatment and PD-1 treatment alone. This data suggested that the combination therapy could not achieve therapeutic synergy in T-cell deficient immunocompromised mice, and therefore T lymphocytes were critical for the antitumor effects of dietary fucoidan.

Next we examined whether the fucoidan pre-treatment would further enhance the therapeutic efficacy of PD-1 blockade. We fed mice with fucoidan containing diet 24 days before tumor inoculation (Figure 4D). We measured the growth of subcutaneous tumors and found that the efficacy of combination therapy was more pronounced. The tumor growth of fucoidan pre-treated mice in the combination therapy group were dramatically slower than the PD-1 monotherapy group (Figure 4E). The sizes and weights (Figures 4F,G) of co-treated tumors were also significantly decreased. Accordingly, FACS demonstrated that combination therapy increased the ratio of CD8^+^/CD4^+^ T cells and percentage of NK cells in spleen (Figure 4H). It is worth noting that compared with the conventional combination therapy, both tumor growth inhibition and T cell activation were more dramatic when mice were pre-treated with the fucoidan diet (Figures 4H vs. 1E). These results suggested that this enhanced anticancer activity may attribute to the activation of whole-body immune responses even before tumor growth. The higher ratio of CD8^+^/CD4^+^ caused by fucoidan pre-treatments suggests that such pre-treatments may intensify the function of PD-1 antibodies by inducing direct changes in T cells.

### Fucoidan Activates the JAK-STAT Pathway and Promotes T Cell Proliferation and Activation

**FIGURE 5:**
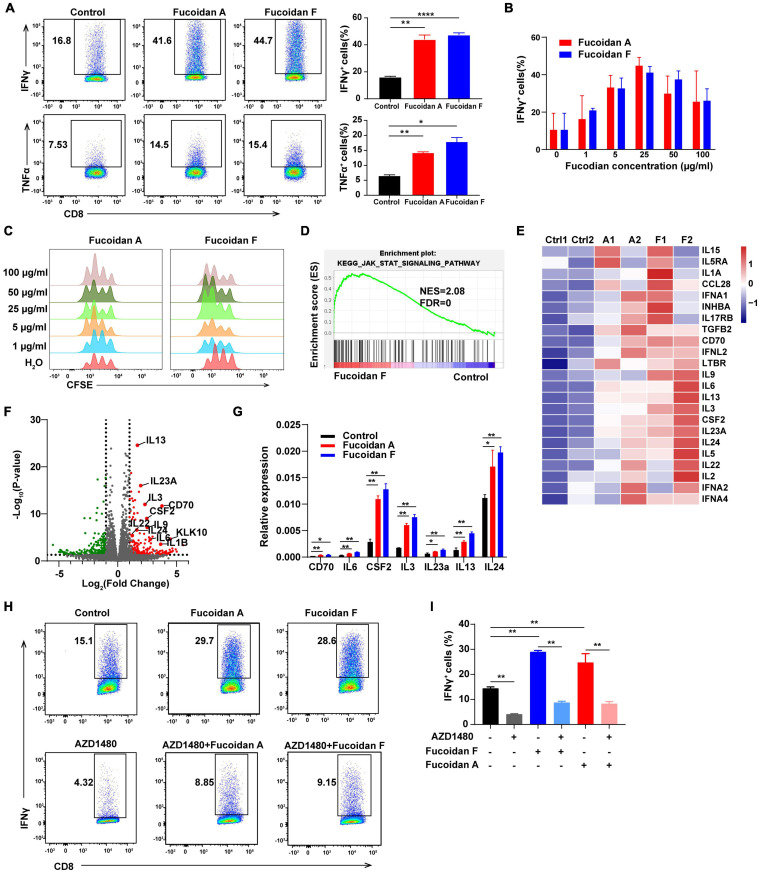
Fucoidan activates the JAK-STAT pathway and promotes T cell proliferation and activation. **(A)** IFNγ and TNFα expression in primary CD8^+^ T cells cultured with anti-CD3/CD28 beads, with or without fucoidan (25 μg/ml) treatments. Numbers adjacent to the outlined areas indicate the frequency of cells expressing IFNγ and TNFα. All data are quantified on the right. **(B)** IFNγ expression levels in CD8^+^ T cells treated by indicated concentrations of fucoidan. **(C)** CFSE assays quantifying the proliferation of CD8^+^ T cells stimulated by indicated concentrations of fucoidan. **(D)** Gene set enrichment analysis (GSEA) of the RNAseq data of fucoidan-treated (10 μg/ml) and control CD8^+^ T cells. **(E)** Heatmap of fucoidan-regulated genes involved in the JAK-STAT pathway. Ctrl, control. A1, A2, two biological replicates of fucoidan A-treated samples. F1, F2, two biological replicates of fucoidan F-treated samples. **(F)** Volcano plots of differentially expressed genes from RNAseq data of fucoidan-treated and control CD8^+^ T cells. The x axis represents log2 of fold changes (FC) of gene expression levels in fucoidan-treated cells relative to control cells, and the y axis represents log10 of corresponding P values. Genes within the JAK-STAT pathway were highlighted. **(G)** qRT-PCR analysis of the mRNA expression of indicated genes in fucoidan-treated and control CD8^+^ T cells. **(H,I)** IFNγ expression in CD8^+^ T cells activated by anti-CD3/CD28 beads, treated with fucoidan (10 μg/ml) and/or AZD1480 (50 nM). Data are representative of three independent experiments. Error bars, mean ± SD. *P < 0.05, **P < 0.01, ***P < 0.001, ****P < 0.0001.

Next, we evaluated whether fucoidan directly stimulates T cell activity. On top of anti-CD3/28 co-stimulatory signals, fucoidan further augmented the effector function of primary CD8^+^ T cells manifested by increased production of cytokines including IFNγ and TNFα (Figure 5A). The amounts of IFNγ were elevated in a dose-dependent manner (Figure 5B). Moreover, fucoidan-treated CD8^+^ T cells underwent cell division more rapidly than PBS-treated control cells, as reflected by the CFSE (carboxyfluorescein succinimidyl amino ester) assay (Figure 5C). Collectively, these data demonstrated that fucoidan directly promotes the expansion and effector function of CD8^+^ T lymphocytes.

To elucidate the molecular mechanisms by which fucoidan stimulates T cells, we performed RNAseq analysis of fucoidan-treated and control CD8^+^ T cells. Gene set enrichment analysis (GSEA) demonstrated that genes within the JAK-STAT pathway were significantly upregulated by fucoidan treatments (Figure 5D). Core genes of this pathway were highlighted in the heatmap and volcano plots shown in Figures 5E,F, and the expression changes of several representative genes, including IL-3, IL-6, IL-13, IL-14, IL-24a, CSF2, and CD70, were further confirmed by qRT-PCR analysis (Figure 5G).

The JAK-STAT pathway is an essential signaling link between cell surface receptors and nuclear transcriptional events. Once activated by extracellular signals, phosphorylated STATs translocate into the nucleus and modulate the expression of multiple target genes, which are critical for T cell activation (Poehlmann et al., 2005; Bousoik and Montazeri Aliabadi, 2018). We next investigated whether fucoidan enhances T cell activity and proliferation through the JAK-STAT pathway. As expected, the JAK1/2 inhibitor AZD1480 impeded the basal activation of CD8^+^ T cells by anti-CD3/28 beads, and fucoidan-mediated stimulatory effects on these T cells were strongly attenuated by AZD1480 administration (Figures 5H,I). These results suggested that fucoidan promotes the proliferation and effector function of CD8^+^ T cells likely via activation of the JAK-STAT pathway.

### Fucoidan Interacts With the T Cell Receptor Complex to Enhance T Cell Activity

**FIGURE 6:**
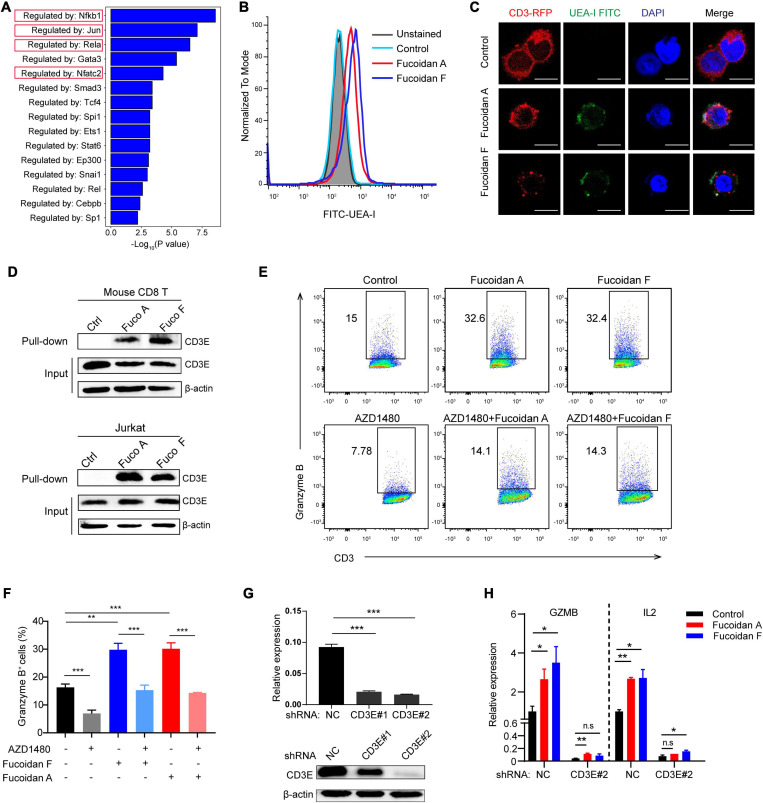
Fucoidan interacts with the T cell receptor complex to enhance T cell activity. **(A)** Genes Ontology (GO) analysis showing the enrichment of specific pathways regulated by indicated transcription factors, based on upregulated hallmark genes in fucoidan-treated CD8^+^ T cells. **(B)** Flow cytometry analysis of CD8^+^ T cells unstained or stained with FITC-labeled Ulex Europaeus Agglutinin I (FITC-UEA-I), supplemented with or without 100 μg/ml fucoidan. **(C)** Representative fluorescent images of Jurkat cells overexpressing RFP-CD3E (red) stained with FITC-UEA-I (green), supplemented with or without 100 μg/ml fucoidan. Nuclei were stained with DAPI (blue). Scale bar, 10 μm. **(D)** Lysates from primary CD8^+^ T and Jurkat cells treated with or without fucoidan were subjected to pull-down assays with Ulex Europaeus Agglutinin I (UEA I) conjugated agarose, followed by immunoblotting analysis with CD3E antibodies. **(E,F)** Granzyme B expression levels in Jurkat cells activated by anti-human CD3/CD28 supplemented with fucoidan (10 μg/ml) and/or AZD1480 (40 μM). **(G)** Knockdown efficiencies of CD3E mRNA and protein using indicated shRNAs in Jurkat cells. **(H)** qRT-PCR analysis of GZMB and IL2 mRNA expression in Jurkat cells transfected with CD3E shRNA#2 or shNC. GZMB, Granzyme B. NC, non-targeted control. Data are representative of two independent experiments. Error bars, mean ± SD. *P < 0.05, **P < 0.01, ***P < 0.001, and n.s., non-significant.

Although fucoidan belongs to natural polysaccharide like starch or glycogen, dietary fucoidan can be absorbed by the human digestive system, which lacks fucoidanase for fucoidan hydrolysis (Silchenko et al., 2013; Atashrazm et al., 2015). Therefore, we reasoned that fucoidan exerts its function by engaging cell membrane receptors. To investigate this, we conducted gene ontology (GO) analysis to explore the potential signaling pathways regulated by fucoidan in CD8^+^ T lymphocytes. GO results unraveled by sentence-based text mining (TRRUST) suggested that the top 5 enriched pathways stimulated by fucoidan are those regulated by transcription factors including *Nfkb*, *Jun*, *Rela*, and *Nfatc2* (Figure 6A). Importantly, the T cell receptor (TCR) signal transduction is triggered by forming a TCR/CD3 complex on the cell surface upon T cell activation, which leads to signal propagations via three major pathways: the Ca^2+^-calcineurin pathway resulting in nuclear translocation of nuclear factor of activated T cells (NFAT), the NFκB signaling pathway resulting in nuclear translocation of REL/NFKB, and the MAPK pathway resulting in actin polymerization and activation of FOS, JUN, activator protein 1 (AP-1) (Gaud et al., 2018). Moreover, the JAK/STAT pathway intertwines with the TCR signaling cascade and potentiates the expression of multiple common target genes, which are essential for the effector function of cytotoxic T cells (Verdeil et al., 2006). Therefore, fucoidan activates all major pathways governed by the TCR signaling, and we speculated that fucoidan may interact with the TCR/CD3 complex in the extracellular space to augment T cell activation. To test this hypothesis, we first explored whether fucoidan associates with T cell membranes, by using a FITC-labeled Ulex Europaeus Agglutinin I (FITC-UEA-I) that specifically recognizes the fucose backbone of fucoidan. Indeed, flow cytometry analysis revealed that FITC-UEA-I fluorescently labeled T cells upon fucoidan treatments (Figure 6B). To confirm that fucoidan binds TCR/CD3 on the T cell surface, we stably overexpressed RFP (red fluorescent protein) labeled human CD3E, a major component of the TCR/CD3 complex (Gil et al., 2002), in Jurkat T cell leukemia cells exhibiting higher transfection efficiencies than primary T cells. Confocal microscopic imaging revealed that supplemented fucoidan, which can be labeled and visualized by FITC-UEA-I, partially colocalized with TCR/CD3 on the cell surface (Figure 6C). Subsequently, we confirmed the interaction between fucoidan and TCR/CD3 with pull-down assays (Figure 6D). Together, these results demonstrated the physical association between fucoidan and the TCR/CD3 complex, which may play important roles in T cell biology.

**FIGURE 7:**
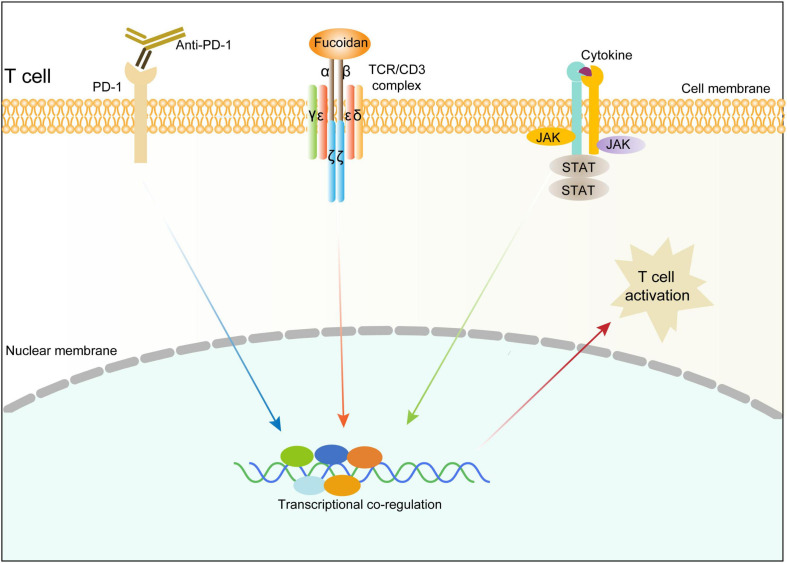
Schematic diagram illustrating the molecular mechanism underlying fucoidan-promoted antitumor immunity.

We next investigated whether the TCR/CD3 complex is required for fucoidan to promote T cell activation. Unlike primary CD8^+^ T cells, Jurkat cells secrete little TNFα and INFγ, yet high amounts of granzyme B in response to TCR/CD3 (Huang et al., 2006; Rasooly et al., 2017) and JAK/STAT signalings upon activation. Indeed, fucoidan supplementation significantly upregulated the expression of granzyme B in Jurkat cells, which were suppressed by AZD1480 co-treatments (Figures 6E,F). Furthermore, we transfected Jurkat cells with shRNAs targeting CD3E or a scramble sequence. Two CD3E shRNAs were effective at the mRNA level, yet only the CD3E shRNA#2 significantly reduced the CD3E protein level (Figure 6G). Therefore, the qRT-PCR assay was applied to evaluate Jurkat T cells with CD3E depletion conferred by shRNA#2. As shown in Figure 6H, CD3E knockdown dramatically suppressed fucoidan-mediated activation of Jurkat cells, as reflected by blunted expression of granzyme B and IL2 upon CD3E depletion. These results suggested that fucoidan binds and functions through the TCR/CD3 complex to enhance TCR-mediated signal transduction and T cell activation (Figure 7).

## Discussion

Diet is the principal method to obtain nutrition for complex systems like human being, and it is well noted that diet intervention for human diseases exhibits characteristics of safety and feasibility. Although the underlying mechanisms remain elusive, an array of dietary approaches have shown powerful antitumor effects (Nencioni et al., 2018). For instance, fasting-mimicking and ketogenic diets have been reported to induce a T-cell dependent tumor growth delay (Lee et al., 2012; Lussier et al., 2016). Furthermore, protein restriction diet can synergize with immunotherapies through reprogramming the tumor associated macrophages (Orillion et al., 2018). Oral supplementation with *Akkermansia muciniphila* and ketone bodies were reported to enhance the efficacy of PD-1 blockade (Routy et al., 2018; Ferrere et al., 2021). Fucoidan in particular is a soluble dietary component that is considered functional food with health benefits (Di Daniele et al., 2017). Oral administration of fucoidan can regulate gut microbiome as a prebiotic (Chen et al., 2019). In addition to be absorbed in the small intestine, fucoidan can also pass through the stomach and small intestine, and produce short-chain fatty acids (SCFAs) when fermented by gut microbiota in the large intestine (Nagamine et al., 2014; Tao et al., 2020). SCFAs are the key energy source of colonocytes, and modulate the function of innate immune cells (Routy et al., 2018; Yao et al., 2020). In this study, we observed moderate increases of splenic NK cells after fucoidan treatments, which may attribute to augmented serum SCFAs.

The immune modulating effects of fucoidan were widely studied. It has been shown that fucoidan enhances the immune responses of NK cells, T cells, macrophages, and DCs (Fitton et al., 2015; Hsu and Hwang, 2019). Our data demonstrated that fucoidan treatments increased the number of NK cells *in vivo*, and promoted DC maturation *in vitro*. However, fucoidan A is more effective than F for inducing NK stimulation, whereas fucoidan F is more effective than A for inducing DC maturation. Unlike NK cells and DCs, mice receiving the fucoidan diet combined with PD-1 therapy exhibit consistent activation of tumor infiltrating CD8^+^ T lymphocytes, which is the only immune cell subset paralleled with tumor shrinkage for two fucoidan species. Nevertheless, T cell activation and tumor inhibition cannot be achieved by fucoidan treatments alone, possibly due to the dampened activity of T cells residing in the immunosuppressive tumor microenvironment (Rabinovich et al., 2007; Thommen and Schumacher, 2018). These suppressed T cells can be partially reinvigorated by PD-1 blockade, explaining the efficacy of combination therapy.

Natural polysaccharides have been reported to play an immunomodulatory role by binding TCR/CD3 or TLR receptors on the T cell surface (Miao et al., 2005; Kabelitz, 2007), and regulate intracellular calcium signals and cytokine production (Zheng et al., 2002; Shuai et al., 2010). However, TLRs are mainly present in the innate immune system and expressed in CD3^+^ T cells at extremely low or non-detectable levels (Applequist et al., 2002). By contrast, T cells in adaptive immunity prefer to receive activation signals through antigen-presenting cells under most circumstances. Although fucoidan was linked to T cells in several studies (Shimizu, 2005; Wang et al., 2019b), its specific role in regulating T cell responses remains unclear. Our RNAseq analysis indicated that fucoidan activates three major pathways downstream of the TCR/CD3 complex, and it is worth noting that TCR/CD3 relies on secondary signaling waves from the JAK/STAT axis to fully sustain its activation capacity. Moreover, we discovered that fucoidan physically interacts with TCR/CD3 on the cell surface and potentiates its activation. The TCR/CD3 complex is consist of multiple building blocks including TCRαβ, CD3ζζ, CD3δε, and CD3γε. The TCRαβ heterodimer is an antigen-binding subunit and lacks inherent signal-transducing domain, whereas CD3 subunits contain the immunoreceptor tyrosine-based activation motif (ITAM) which could be phosphorylated and responsible for the signal transduction from TCR to intracellular pathways (Pitcher and van Oers, 2003; Kuhns et al., 2006; Xu et al., 2020). To test whether the TCR/CD3 complex is required for fucoidan to stimulate T cells, we suppressed CD3E expression using targeted shRNAs, as the encoded CD3ε is critical for T cell activation (Gil et al., 2002). CD3ε depletion significantly inhibits fucoidan-mediated T cell activation, combined with the fact that fucoidan physically associates with TCR/CD3, demonstrating that fucoidan exerts it antitumor function by engaging the TCR/CD3 complex and promoting cytotoxic T cell activities.

In conclusion, we discovered that fucoidan as a dietary integradent can coordinate with PD-1 therapy and strongly potentiate its antitumor effects. Mechanistically, fucidin enhances the proliferation and cytokine production of tumor infiltrating CD8^+^ T cells through binding to the TCR/CD3 complex. Our study provides tangible evidence to underscoring orally-delivered fucoidan as a synergistic anti-cancer agent with immunotherapy.

## Data Availability Statement

The RNA-seq data generated in this study have been deposited in the Sequence Read Archive with the accession number PRJNA743936.

## Ethics Statement

The animal study was reviewed and approved by Sun Yat-sen University laboratory animal center.

## Author Contributions

J-XB, CP, GY, PZ, and BL conceived the study and analyzed the data. JY, XY, WP, MW, JZ, ZF, XZ, YL, JD, YW, and YJ designed and performed the experiments. JY and BL wrote the manuscript. All authors contributed to the article and approved the submitted version.

## Conflict of Interest

The authors declare that the research was conducted in the absence of any commercial or financial relationships that could be construed as a potential conflict of interest.

## Publisher’s Note

All claims expressed in this article are solely those of the authors and do not necessarily represent those of their affiliated organizations, or those of the publisher, the editors and the reviewers. Any product that may be evaluated in this article, or claim that may be made by its manufacturer, is not guaranteed or endorsed by the publisher.

## Footnotes

1
http://metascape.org/
